# Genomic profiling of *Pasteurella multocida* strains isolated from ISA Brown (*Gallus gallus domesticus*) in Bangladesh

**DOI:** 10.1128/mra.00048-26

**Published:** 2026-04-01

**Authors:** Md. Alimul Islam, Md. Enamul Haque, Md. Iftehimul, Mohammad Sadekuzzaman, Tanbin Rubaiya Islam, Md. Sojon Miah, Sajedul Hayat, Susmita Karmakar, Muhammad Tofazzal Hossain

**Affiliations:** 1 Department of Microbiology and Hygiene, Bangladesh Agricultural University 54492, Mymensingh, Bangladesh; 2 Institute of Biotechnology, Bangladesh Agricultural University 54492, Mymensingh, Bangladesh; 3 Central Disease Investigation Laboratory, Department of Livestock Services, Dhaka, Bangladesh; Indiana University Bloomington, Bloomington, Indiana, USA

**Keywords:** *Gallus gallus domesticus*, *Pasteurella multocida*, poultry farming, fowl cholera, WGS

## Abstract

Poultry farming faces fowl cholera caused by *Pasteurella multocida*, yet genomic data are limited. Isolates from ISA Browns were identified using matrix-assisted laser desorption/ionization time-of-flight (MALDI-TOF) and sequenced (8.6, 1.4, and 1.3 M reads). *De novo* assemblies revealed genome sizes of ~2.4–2.5 Mb and guanine or cytosine (GC) content of ~40%.

## ANNOUNCEMENT

Poultry farming is a vital industry for the economy and food security in Bangladesh. The country possesses a significant population of domestic and commercial layer chickens like ISA Brown (*Gallus gallus domesticus*), which are essential sources of meat and eggs (1). The frequency of infectious diseases, such as fowl cholera (*Pasteurella multocida*), presents a substantial threat to this business. Nevertheless, as regards the significance of *P. multocida* in causing epidemics, there exists a dearth of extensive genetic data regarding the strains prevalent in Bangladesh (2). *P. multocida* isolated from fowl cholera suspected heart samples was collected from layer chickens in the Mymensingh (24.7460° N, 90.4179° E), Gazipur (23.9905° N, 90.3877° E), and Tangail (24.2515° N, 89.9198° E) in Bangladesh. The collected 1 g of samples was incubated overnight at 37°C in Luria-Bertani (LB) broth (Oxoid, Thermo Fisher Scientific, UK). A loopful of the inoculum from the LB culture was streaked onto blood agar base (Oxoid, Thermo Fisher Scientific, UK) enriched with 5% sheep blood and incubated at 37°C for 24–48 h. Colonies of *P. multocida* were identified based on their colonial morphology and characteristic bipolar staining. Pure cultures of *P. multocida* were obtained by selective subculturing guided by colony characteristics. The isolated samples were further analyzed by the matrix-assisted laser desorption/ionization time-of-flight (MALDI-TOF) (3) technique according to the manufacturer’s protocol in the Quality Control Laboratory, Savar, Dhaka, Bangladesh.

A single colony of each strain (FC1, FC2, and FC3) was grown in LB broth at 37°C for 10 h, and genomic DNA was extracted using the QIAamp DNA Mini Kit (Qiagen, Germany) following the manufacturer’s instructions, and Illumina libraries were prepared using the Illumina DNA Prep Kit (Illumina, Inc., San Diego, CA) following the manufacturer’s instructions for next-generation sequencing. The libraries were then sequenced on the Illumina NovaSeq 6000 platform and generated 8.6, 1.4, and 1.3 million paired-end reads for FC1, FC2, and FC3. Raw sequencing reads were first subjected to quality assessment using FastQC v0.12.1 to evaluate base quality scores, GC content, read length distribution, and potential sequencing artifacts (4). Adapter sequences, low-quality bases, and ambiguous reads were then removed using fastp v0.24.0, ensuring high-quality data for downstream analyses (5). The filtered reads were assembled *de novo* using SKESA v2.2, a genome assembler optimized for bacterial whole-genome sequencing data (6). Assembly quality was evaluated with QUAST v5.3.0, which provided summary statistics including genome size, number of contigs, N50 values, guanine or cytosine (GC) content, and overall assembly integrity (7). Assembly accuracy was further assessed using CheckM v1.2.3 (8), which estimates genome completeness and contamination based on lineage-specific marker genes. To confirm taxonomic identity and genomic relatedness, average nucleotide identity (ANI) was calculated using FastANI (9) by comparing the assembled genomes to a reference strain (accession: GCF_002073255.2). Genome annotation of isolates FC1, FC2, and FC3 was performed using the National Center for Biotechnology Information (NCBI) Prokaryotic Genome Annotation Pipeline (PGAP) v6.9, enabling the prediction of coding sequences, RNA genes, and functional elements (10). Functional genomic analysis included the identification of antibiotic resistance genes (ARGs) using ResFinder (11) and the detection of virulence factor genes (VFGs) through the Virulence Factor Database (VFDB) (12). All analyses were conducted using default software parameters to ensure consistency and reproducibility, unless otherwise stated.

The sequencing generated 8.6 million reads for FC1, 1.4 million reads for FC2, and 1.3 million reads for FC3. These high-quality reads were assembled into complete genomes, resulting in average coverages of 1,018× for FC1, 141× for FC2, and 146× for FC3, ensuring high confidence in the assemblies. Detailed assembly metrics, including the number of contigs, N50 values, genome sizes, GC content for each isolate, and so on, are summarized in Table 1 across all three isolates.

**TABLE 1:** Information regarding genome sequence of *P. multocida* strains isolated from the fowl cholera suspected ISA Brown (*Gallus gallus domesticus*) in Bangladesh

Parameters	*P. multocida* FC1	*P. multocida* FC2	*P. multocida* FC3
GenBank accession	GCA_049348065	GCA_049348105	GCA_049348145
Nucleotide accession	DBHLZZ000000000	DBHLZY000000000	DBHLZW000000000
Contigs	44	41	51
Genome length (bp)	2,538,122	2,575,784	2,479,798
GC content (%)	40.40	40.45	40.49
Contig L50	4	4	4
Contig N50	289,241	289,241	163,798
Genes (total)	2,460	2,503	2,399
CDSs (total)	2,400	2,444	2,333
Genes (coding)	2,377	2,423	2,310
CDSs (with protein)	2,377	2,423	2,310
Genes (RNA)	60	59	
rRNAs	2, 2, 1 (5S, 16S, 23S)	2, 2, 1 (5S, 16S, 23S)	3, 3, 3 (5S, 16S, 23S)
Complete rRNAs	1, 1 (5S, 23S)	1, 1, 1 (5S, 16S, 23S)	2, 1, 1 (5S, 16S, 23S)
Partial rRNAs	1, 2 (5S, 16S)	1 (5S)	1, 2, 2 (5S, 16S, 23S)
tRNAs	51	51	53
ncRNAs	4	4	4
Pseudo genes (total)	23	21	23
CDSs (without protein)	23	21	23
Pseudo genes (ambiguous residues)	0 of 23	0 of 23	0 of 23
Pseudo genes (frameshifted)	9 of 23	8 of 23	9 of 23
Pseudo genes (incomplete)	9 of 23	8 of 23	9 of 23
Pseudo genes (internal stop)	10 of 23	9 of 23	10 of 23
Pseudo genes (multiple problems)	4 of 23	3 of 23	4 of 23
Completeness	100	100	100
Contamination	4.7	7.1	4.7
Genome quality	Good	Good	Good
ANI	98.55	98.55	98.57
Pathogenicity	0.826	0.834	0.826
AMR genes	7	7	7
Virulence genes	3	3	3

## Data Availability

All raw *Pasteurella multocida* genome sequences have been deposited in NCBI under BioProject accession number PRJNA514245. The corresponding Sequence Read Archive (SRA) accession numbers are SRR32921853, SRR32921852, and SRR32921851, and the associated BioSample accessions are SAMN47638445, SAMN47638446, and SAMN47638447.
